# Human-machine interface for two-dimensional steering control with the auricular muscles

**DOI:** 10.3389/fnbot.2023.1154427

**Published:** 2023-06-05

**Authors:** Daniel J. L. L. Pinheiro, Jean Faber, Silvestro Micera, Solaiman Shokur

**Affiliations:** ^1^Division of Neuroscience, Department of Neurology and Neurosurgery, Neuroengineering and Neurocognition Laboratory, Escola Paulista de Medicina, Universidade Federal de São Paulo, São Paulo, Brazil; ^2^Translational Neural Engineering Lab, Institute Neuro X, École Polytechnique Fédérale de Lausanne, Geneva, Switzerland; ^3^Neuroengineering Laboratory, Division of Biomedical Engineering, Instituto de Ciência e Tecnologia, Universidade Federal de São Paulo, São José dos Campos, Brazil; ^4^Department of Excellence in Robotics and AI, Institute of BioRobotics Interdisciplinary Health Center, Scuola Superiore Sant'Anna, Pisa, Italy

**Keywords:** Neuroprosthetics, human-machine interface, auricular muscle, motor decoding, steering control

## Abstract

Human-machine interfaces (HMIs) can be used to decode a user's motor intention to control an external device. People that suffer from motor disabilities, such as spinal cord injury, can benefit from the uses of these interfaces. While many solutions can be found in this direction, there is still room for improvement both from a decoding, hardware, and subject-motor learning perspective. Here we show, in a series of experiments with non-disabled participants, a novel decoding and training paradigm allowing naïve participants to use their auricular muscles (AM) to control two degrees of freedom with a virtual cursor. AMs are particularly interesting because they are vestigial muscles and are often preserved after neurological diseases. Our method relies on the use of surface electromyographic records and the use of contraction levels of both AMs to modulate the velocity and direction of a cursor in a two-dimensional paradigm. We used a locking mechanism to fix the current position of each axis separately to enable the user to stop the cursor at a certain location. A five-session training procedure (20–30 min per session) with a 2D center-out task was performed by five volunteers. All participants increased their success rate (Initial: 52.78 ± 5.56%; Final: 72.22 ± 6.67%; median ± median absolute deviation) and their trajectory performances throughout the training. We implemented a dual task with visual distractors to assess the mental challenge of controlling while executing another task; our results suggest that the participants could perform the task in cognitively demanding conditions (success rate of 66.67 ± 5.56%). Finally, using the Nasa Task Load Index questionnaire, we found that participants reported lower mental demand and effort in the last two sessions. To summarize, all subjects could learn to control the movement of a cursor with two degrees of freedom using their AM, with a low impact on the cognitive load. Our study is a first step in developing AM-based decoders for HMIs for people with motor disabilities, such as spinal cord injury.

## 1. Introduction

In recent years, various motor Neuroprosthetics (NP) have been proposed to reduce the burden caused by spinal cord injury (Shokur et al., 2021). For example, surface functional electrical stimulation of the muscles (Ho et al., 2014), exoskeletons (Tucker et al., 2015; Baud et al., 2018; Benabid et al., 2019), electrical stimulation of the spinal cord (Greiner et al., 2021), the peripheral nerves (Badi et al., 2021) allows to re-mobilize the paralyzed body part (Losanno et al., 2023), silent speech recognition (Cai et al., 2021; Wu et al., 2021, 2022). But, while these assistive devices allow significant improvement in motor functions, efficient technologies still need to permit their voluntary control.

Brain-machine interfaces (BMIs) have sometimes been proposed as the ultimate solution for voluntary motor decoding (Lebedev and Nicolelis, 2017; Salahuddin and Gao, 2021). Despite their great potential, BMIs should first overcome many non-trivial challenges before their wider adoption, including the improvement of surgery safety (Loeb and Richmond, 2021), the long-term stability of the electrodes (Chestek et al., 2011), decoding (Hill et al., 2012; Tankus et al., 2014; Jin et al., 2019), among others (Freire et al., 2011; Rapeaux and Constandinou, 2021). This technique has also been explored in a non-invasive manner and, although good results can be found, in general, it does not respond that well in a daily environment due to the quality of the record and the need for constant calibration (Douibi et al., 2021; Värbu et al., 2022).

Instead, a different approach can target patients' preserved muscular functions, typically in the face and the head, to decode their intentions and use them as input controllers. Various input mechanisms have been studied to this end, including head orientation (Evans et al., 2000; LoPresti et al., 2002), tongue movement (Andreasen Struijk, 2006; Mohammadi et al., 2021), neck (Williams and Kirsch, 2008, 2016), and facial muscles and kinematics (Huang et al., 2006; Cler and Stepp, 2015; Galán et al., 2016). Others have based their decoding on the auricular muscles (AMs) (Perez-Maldonado et al., 2010; O'Meara et al., 2019). The latter has the advantage that the AMs is a vestigial muscle that is normally not used and is, therefore, an excellent candidate to enhance remaining functions without hindering the preserved ones. Perez-Maldonado et al. (2010) tested a two-dimensional cursor control using the AM decoding through a linear combination of surface electromyography (sEMG) signal's frequency band activation. While this approach showed encouraging results, it lacked a simple strategy to stop at the desired point and needed 15 sessions to train the subjects to master the control. The same group introduced, in 2019, a more robust approach based on a 1D translation control with the AM decoder plus an automatic rotation. To stop moving, the subject needed to relax the muscle contraction. While the approach improved the stopping strategy, the control is slower, and changes in movement orientation are rather complex. Schmalfuß et al. (2016) used minimally invasive EMG electrodes implanted in the posterior auricular muscles of healthy and SCI individuals to learn to control a wheelchair.

Here, we propose a novel decoding and training strategy leveraging standard non-invasive surface EMG electrodes bilaterally placed on the superior auricular muscle. The central aspect of our work is a control strategy that facilitates the moving and the stopping of the cursor and the crafting of a training protocol that reinforces the orthogonalization between AM contraction and facial expression.

Following 5 sessions of 30 min training we found that all participants (5 healthy subjects) could master the tasks with the virtual cursor, with a higher success rate and more reliable control. The results presented here open the way for implementing AM for steering control, either in virtual or non-virtual solutions, for people with motor disabilities.

## 2. Methods

### 2.1. sEMG recording and decoding strategy

**Figure 1:**
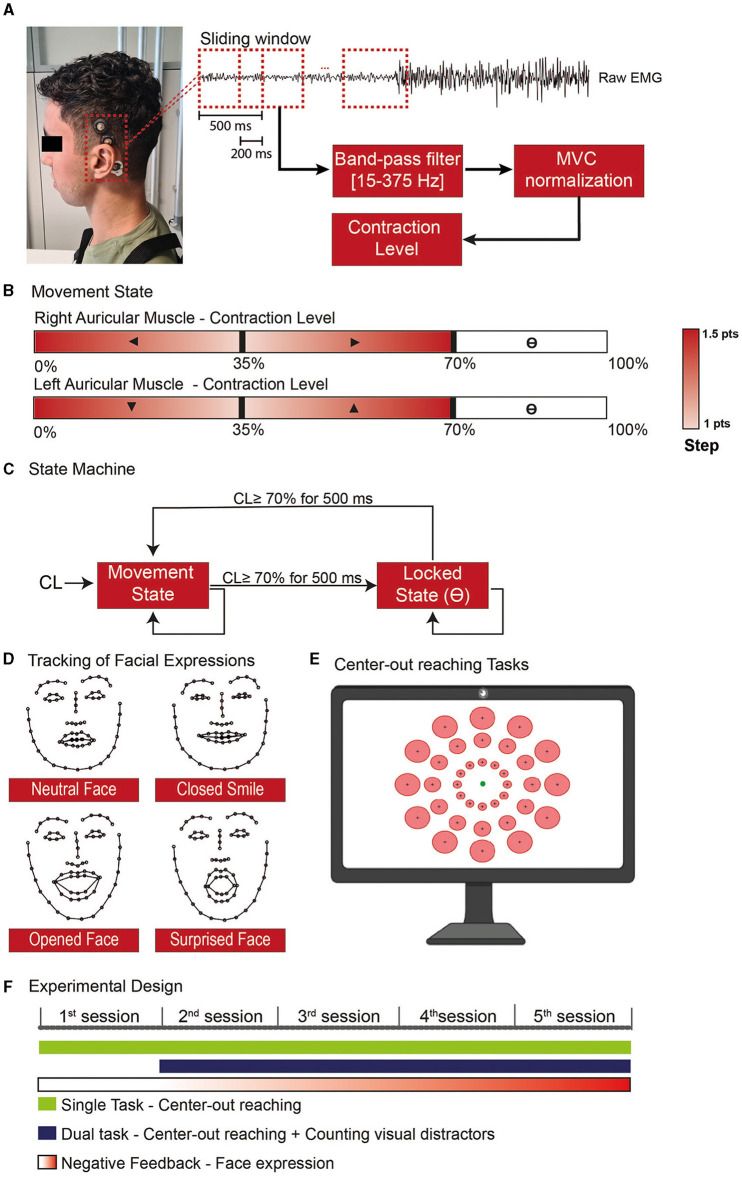
Experimental steps for the use of the Auricular muscle to control a cursor in a 2D paradigm. **(A)** sEMG activity was recorded on the superior AM using cup electrodes associated with a conductive paste to improve signal quality and electrode stability. **(B)** Whenever the user was in the movement state, each ear would control one of the cartesian axes, and the AM contraction level would give the movement direction. **(C)** The AM contraction level was used to feed a state machine to determine whether the end-effector should be in a movement or locked state, allowing the user to independently stop in a specific position with one/both axis-direction, undetermined block the cursor position, or assume the control back without the aid of an external command. **(D)** The level of facial expressions was tracked with the use of a network trained to recognize 68 face landmarks. To train the model used to distinguish the neutral and non-neutral face, at the beginning of each session the subjects had to do specific expressions. **(E)** Both the single and dual tasks performed during the training consisted of center-out reaching tasks where one of the circular targets (red) was presented and the subject would have the 20s to move the cursor (green circle) toward the target and stop inside. **(F)** The experimental design was divided into 5 training sessions where the subject would be requested to use the AM to perform specific tasks with progressive negative feedback according to the level of facial.

sEMG signal was recorded from both superior auricular muscles (Figure 1A) using surface Ag/AgCl cup electrodes with a conductive paste to improve the conductance and help the electrode stick to a region that generally has hair. The reference and ground electrodes were placed on the mastoid bones. The electrodes were connected to the Gravepine Ripple processor for bio-signal amplifications, real-time acquisition, and communication, with a sample rate of 2 kHz, a band-pass filter between 15–375 Hz, and a 50/100/150 notch filter. The control strategy was based on the contraction level of the two AMs. We measure the root mean square of the rectified sEMG signal using a sliding window of 500 ms, updated every 200 ms.

In our implementation, the X-axis was controlled by the right AM, whilst the Y-axis was controlled by the left one (Figure 1B). Specifically, a contraction below 35% of the Maximum Voluntary Contraction (MVC) of the right AM triggered movement toward the left, and a contraction in the 35–70% MVC range moved the cursor toward the right (same for the up/down control with the left AM). The cursor step in a given direction variated between 1 and 1.5 points, in a squared field of 100 × 100 points. The small step was obtained with MVC close to 35%. The steps were linearly increased to 1.5 when the contraction level (CL) went to 0% MVC or, respectively, to 70%.

We used the high contraction range (70–100%) to toggle a locking mechanism on the corresponding dimension (Figure 1C): to transition between the movement and locked states, the users should maintain a strong contraction for 500 ms.

### 2.2. Face expression detection

To force participants to contract their AM while avoiding open facial movements, we tracked their facial expressions during the sessions. Given the proximity and overlap with facial muscles, such as the temporalis, frontalis, and muscles in the infra and supraorbital region of the month (von Arx et al., 2018), we target to detect movements that involve the use of smiles and eyes in the expressions (Figure 1D).

The subjects were sitting in a chair in front of a computer screen and a webcam (Aukey, 1080P, 30 FPS). To quantify the level of facial expression, we used a neural network proposed by Sagonas et al. (2016) able to identify 68 face landmarks. With these landmarks, we train a model to classify when the subject was doing a neutral or non-neutral face. To train the model, we started each session with a training phase where the subjects were requested to do five types of facial expressions: (1) resting face, (2) Closed mouth smile, (3) Open mouth smile, (4) Large smile, and (5) Surprised face. Each expression was maintained for 10 seconds.

We extracted the width and length of face structures (eyebrows, eyes, nose, mouth, and face contour) from the landmarks identified on the frames and performed a principal component analysis to select the components that represent at least 95% of the explained variance. These components were used to train a linear discriminant analysis (LDA) to discriminate between neutral and non-neutral facial expressions; we describe as *FE* the level of facial expression ranging between 0 (neutral face) to 1 (full detection of a given facial expression) as given by the LDA classifier (subject's model performance in Supplementary Figure S1). This value was used as a penalty score for the cursor speed.

### 2.3. Experimental design

Five healthy young adults (mean ± std: 26.20 ± 3.70 years), naive to the training protocol, were included in the study. The study was approved by the Commission Cantonale d'éthique de la recherche Genève (BASEC-ID: 2019-02176). All participants provided written consent to participate in the study. Participant 5 is one of the co-authors of the current study; he was not trained to perform AM contraction before the protocol described here. All pilot tests were done with different participants excluded from this study.

Each session started with a baseline recording to estimate the Maximum Voluntary Contraction (MVC) for each AM. Participants were instructed to relax and contract for 5 seconds, 3 times. We next calculated the root mean square to set the minimum and maximum AM contraction.

The training consisted of single and dual tasks and lasted 20–30 min. The main protocol was a center-out reaching task with 36 possible circular targets, all with the same index of difficulty (ID = 2) as defined by Fitt's Law (Fitts, 1954) (Figure 1E).


$$\begin{array}{l} ID=Log_{2}\left(2D/W\right) \end{array}$$

where D is the target's distance from the starting point and W its radius.

For a trial to be considered successful, the subject had to stop inside of the target and stay there for at least 2 seconds. The timeout was set at 20 seconds. An auditory cue informed the participant of the outcome of the trial.

During the dual task, random visual distractors with the twice the size of the cursor appeared for 100–150 milliseconds every 1.28 ± 0.08 seconds (mean ± standard error) (Supplementary Video S1). The participants had to perform the same center-out reaching as to the single task and count the number of visual distractors presented during the trial and report the number of observed distractors orally at the end of each trial.

Each Training block lasted 18 trials. On the first experimental day, the subject performed two single-task blocks (2 blocks of 18 trials); from sessions 2 to 5, they performed one single task and one dual-task every day (Figure 1F).

The Cursor Speed was defined by both the contraction level (*CL*, measured as a percentage of the MVC) and the level of facial expression *FE* (added as a penalty):


$$\begin{array}{l} Cursor\;Speed\;=\;S{\;}^{*}\left(1-PC^{*}FE\right)\;\\ \text{where }S\;=\;CL{\;}^{*}\left(-50/35\right)\;+\;1.5,\;if\;CL\;\leq \;0.35\\ S=CL^{*}\left(50/35\right)+0.5,\;if\;CL>0.35\;and\;CL\\ \leq 0.7 \end{array}$$

and *PC* is a penalty coefficient for facial expressions. PC was set to 0 on the first day (participants were allowed to use facial expressions) and increased to 0.33 on the next day and to 0.66 on day 3. We set PC to 1 for the last 2 days. This incremental negative feedback allowed the subjects to experiment with different control strategies that avoided facial expressions.

### 2.4. Performance evaluation

To assess participants' performance, we report the following measurements:

(i) Success rate per session.

(ii) The final distance to the target. For each trial, the distance between the center of the target and the cursor.

(iii) The path straight deviation, defined as


$$\begin{array}{l} PathStrDev\;=\;\frac{1}{n}\sum_{i=1}^{n}d\left(P_{i},\bar{\text{a}}\right) \end{array}$$

where in *n* is the number of steps contained in the trajectory, $\bar{\text{a}}$ is the vector that represents the shortest trajectory to the target and *P*_*i*_ a given point on the trajectory, and *d* is the euclidean distance between the point and the trajectory $\bar{\text{a}}$.

(iv) The path efficiency, defined as


$$\begin{array}{l} PathEff\;=\;\frac{\sum_{i=1}^{n}d\left(P_{i},P_{i-1}\right)}{\left\|\bar{\text{a}}\right\|}\; \end{array}$$

(v) Trial execution time. In trial failure, the execution time is set to the timeout (=20s).

(vi) Cursor speed is measured in *pts/s* for each trial. In the case of a successful trial, we removed the samples from the last 2 seconds of the trial (where the participants had to stop inside the target).

For the facial expressions, we calculate the mean level of facial expression throughout the trials and the percentage of each trial where we detected some facial expressions.

For the cognitive load assessment, we applied the NASA Task Load Index (TLX) (Stanton et al., 2017), aiming to evaluate six different domains related to the task execution with a 9-Likert scale. We also evaluated the participants' mean error in detecting the visual distractors during the dual task as follows:


$$\begin{array}{l} error_{distractors\;}=\;\frac{1}{n}\overset{n}{\underset{i}{\sum }}\left(\frac{\left|\left(real_{i}\;-\;detected_{i}\right)\right|}{real_{i}}\right)\; \end{array}$$

where *n* represents the number of trials, *real* is the correct number of distractors in a given trial and *detected* is the number reported by the participant.

### 2.5. Statistical analysis

We tested the normality of the data with the Shapiro-Wilk test, and given the rejection of the null hypothesis, we proceeded with the non-parametric test for further analysis. We used Friedman's test to evaluate the statistical significance of the performance metrics calculated in all trials over the training sessions and between tasks. To test if there were significant effects on the success rate and questionnaires, we used the Kruskal-Wallis, with Tukey-Kramer correction as a post-doc. The significance level for all the analyzes was a = 0.05. The results are represented by median and median absolute deviation. All the comparisons between single and dual tasks were made with a two-sided paired Wilcoxon signed rank test.

## 3. Results

### 3.1. Control performance

**Figure 2:**
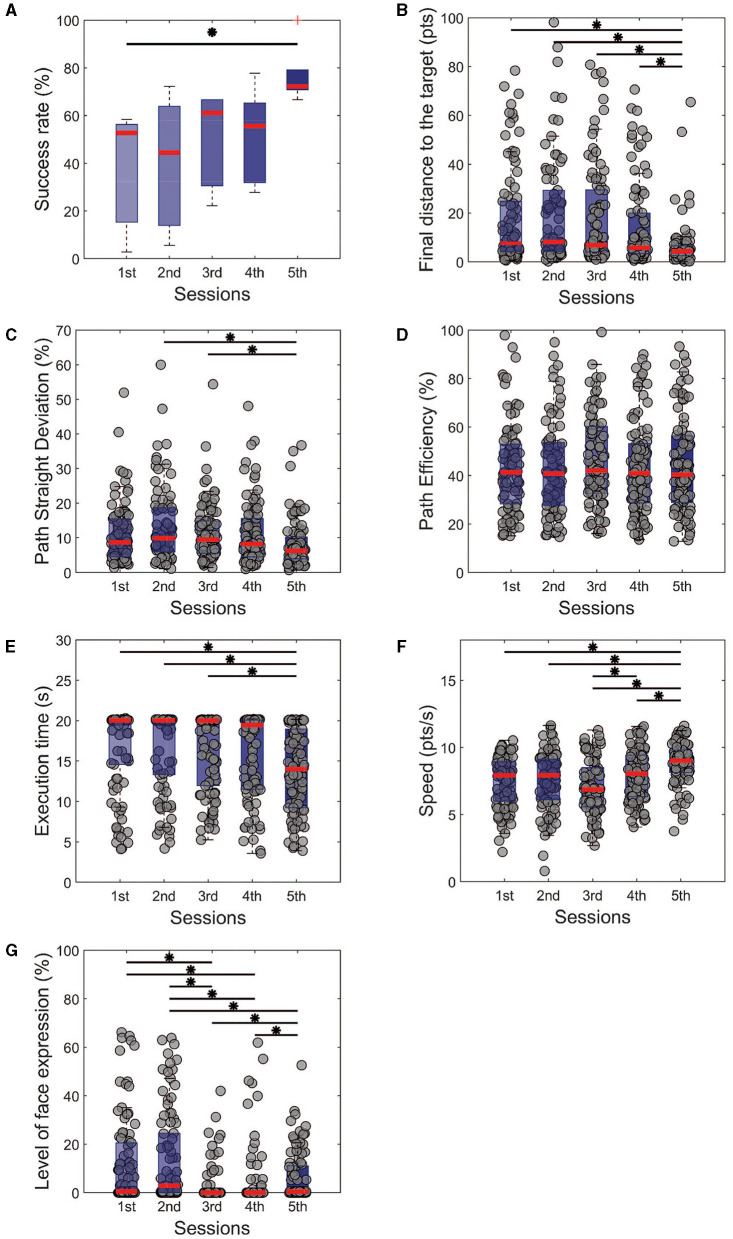
Performance metrics for the single task of all training sessions. It is noticeable that the subjects managed to improve their control in terms of **(A)** success rate and **(B)** precision with the final distance from the target. From the perspective of trajectories, **(C)** the path was getting straighter with the training, even without being more **(D)** efficient. **(E)** The execution time was reduced over the days, with crescent modulation of the speed **(F)**. We also observe a significant reduction in the level of facial expressions **(G)** with the sessions. Kruskal-Wallis test with Tukey-Kramer *post-hoc* correction was used to test the success rate. For trial-level analysis **(B–G)**, repeated measures of Friedman's test were used, with the same *post hoc* correction. Statistical significance among the sessions was considered for *p* < 0.05 (*).

On the single task, we observed a significant improvement from the first training session to the fifth one, associated with a higher success rate (Figure 2A, Success rate 5th session = 72.22 ± 6.67%, 1st session = 52.78 ± 5.56%, *p* < 0.05, χ2 = 9.93) and higher precision (Figure 2B, distance to the target at the end of the trial = 19.61 ± 11.30 pts for the first session and 6.96 ± 2.05 pts for the fifth, *p* < 0.001, χ2 = 34.73). While the path efficiency did not significantly improve throughout the training (Figure 2C, *p* = 0.59, χ2 = 2.80), we found a significant improvement in the path straight deviation (Figure 2D, *p* < 0.001, χ2 = 15.86). We can note that the trials' duration in the last session was also shorter than the first three training sessions (Figure 2E); similarly, participants were significantly faster in performing the task on the final experimental day (Figure 2F). This effect is, most probably, related to the lack of incremental penalty due to facial expression in the last session. We believe that from the 2nd to the 4th training session the subjects always have to adapt a bit their control strategy in order to control the cursor and improve their performance. This adaptation is not needed in the last training session since there is no new penalty score due to facial expressions.

This is particularly interesting, considering that a negative penalty was given throughout the sessions when the participants had open facial expressions. The measurement of the level of facial expressions showed a decrease between the first and third day (1st session: 16.22 ± 12.71%, 3rd session: 0.50 ± 0.50%, *p* < 0.001, χ2 = 100.08) and maintained low for the following days. The results mentioned above show that from different aspects, the cursor control was improved toward a straighter trajectory, a faster control, and closer to the target position.

**Figure 3:**
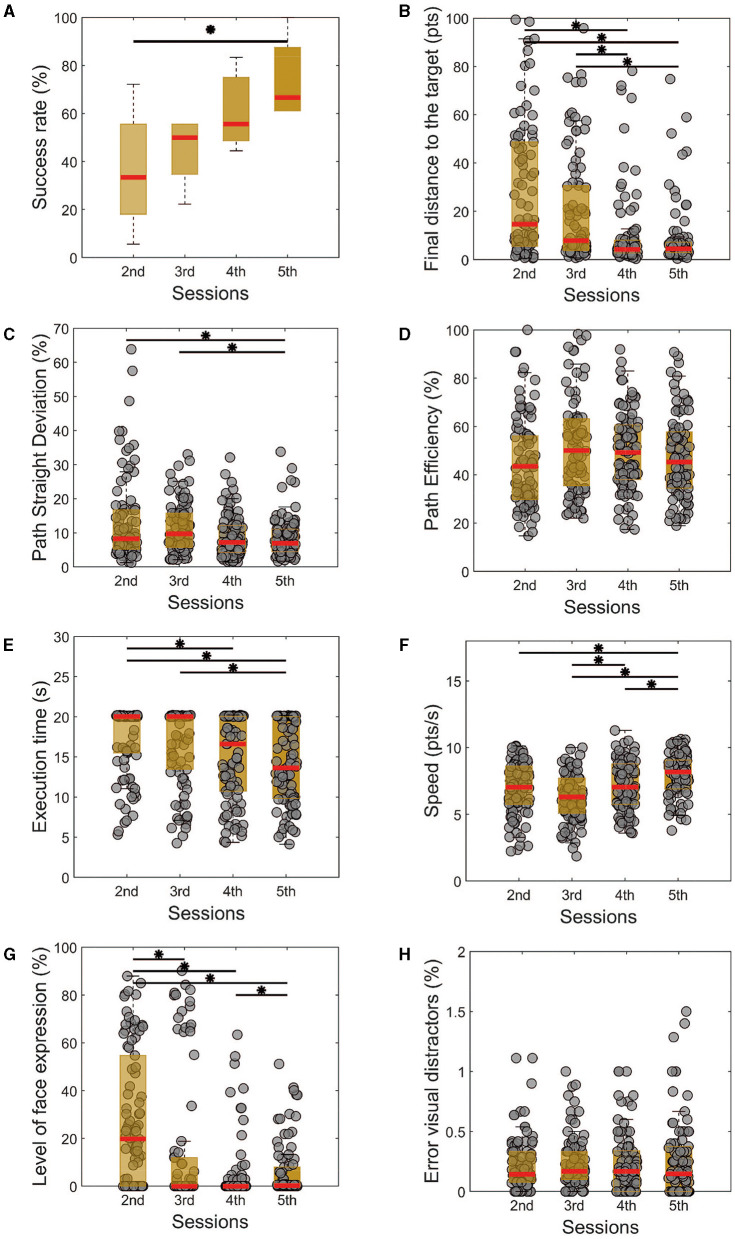
Performance metrics for the dual task of all training sessions. Similarly, as the single task, there is a noticeable improvement in the success rate **(A)**, followed by a reduction in the final distance from the target **(B)**, path straight deviation **(C)**, execution time **(E)**, level with facial expressions **(G)**. The path efficiency **(D)** does not show a significant difference throughout the sessions. **(H)** The error between the perceived and real visual distractors shows no differences among the sessions but is always in a very low percentual. Kruskal-Wallis test with Tukey-Kramer *post-hoc* correction was used to test the success rate. For trial-level analysis **(B–H)**, repeated measures of Friedman's test were used, with the same *post hoc* correction. Statistical significance among the sessions was considered for *p* < 0.05 (*).

Similarly, we observed an improvement in the success rate in the dual task (Figure 3A, 5th session = 66.67 ± 5.56%, 2nd = 33.33 ± 16.67, *p* < 0.05, χ2 = 8.57), from the second experimental day to the last one, which is also reinforced by a reduction in the final distance from the target (Figure 3B, 5th session = 8.37 ± 1.59 pts, 2nd = 25.85 ± 3.44%, *p* < 0.001, χ2 = 41.59). Unlike the single task, the effect on the final distance can already be observed on the fourth experimental day.

Analyzing the path efficiency in Figure 3C, we do not observe significant changes in the cursor-controlled trajectory (*p* = 0.09, χ2 = 6.41). A significant reduction in the path straight deviation was already observed for the last training sessions (Figure 3D, *p* < 0.001, χ2 = 12.92). We also observed that the time needed to finish the task was reduced on the last day compared to the first two sessions (Figure 2E, *p* < 0.001, χ2 = 18.87), and the control speed was improved from the third experimental day to the last one (Figure 3F, *p* < 0.001, χ2 = 31.81).

In accordance with the single task, here we can see that the facial expression during the control improved from the first to the last day in the evaluated metric (Figure 3G, *p* < 0.001, χ2 = 56.12). Particularly, the better performance on this aspect happened on the fourth day of the experiment, which can represent a bit of the need for more training and regularity to properly evaluate this independence of the AM contraction from the other facial muscles.

In summary, the results showed that all participants were able to successfully control the cursor in both single and dual-task scenarios over the course of five training sessions. In the final session of the single task, all participants achieved a correct target hit rate of over 66.67% (Supplementary Table S1) with an average final distance from the target of 6.45+/−1.21 pts (Supplementary Table S2). Similarly, in the final session of the dual task, all participants achieved a correct target hit rate of over 61.11% (Supplementary Table S3) with an average final distance from the target of 8.85+/−1.56 pts (Supplementary Table S4). In all these cases the effect size was estimated through the Cohen's d index and the results show a high effect of the statistics. These findings suggest that even with a small sample size, the participants consistently improved their performance and achieved a good level of control over the human-machine interface.

### 3.2. Cognitive load assessment

**Figure 4:**
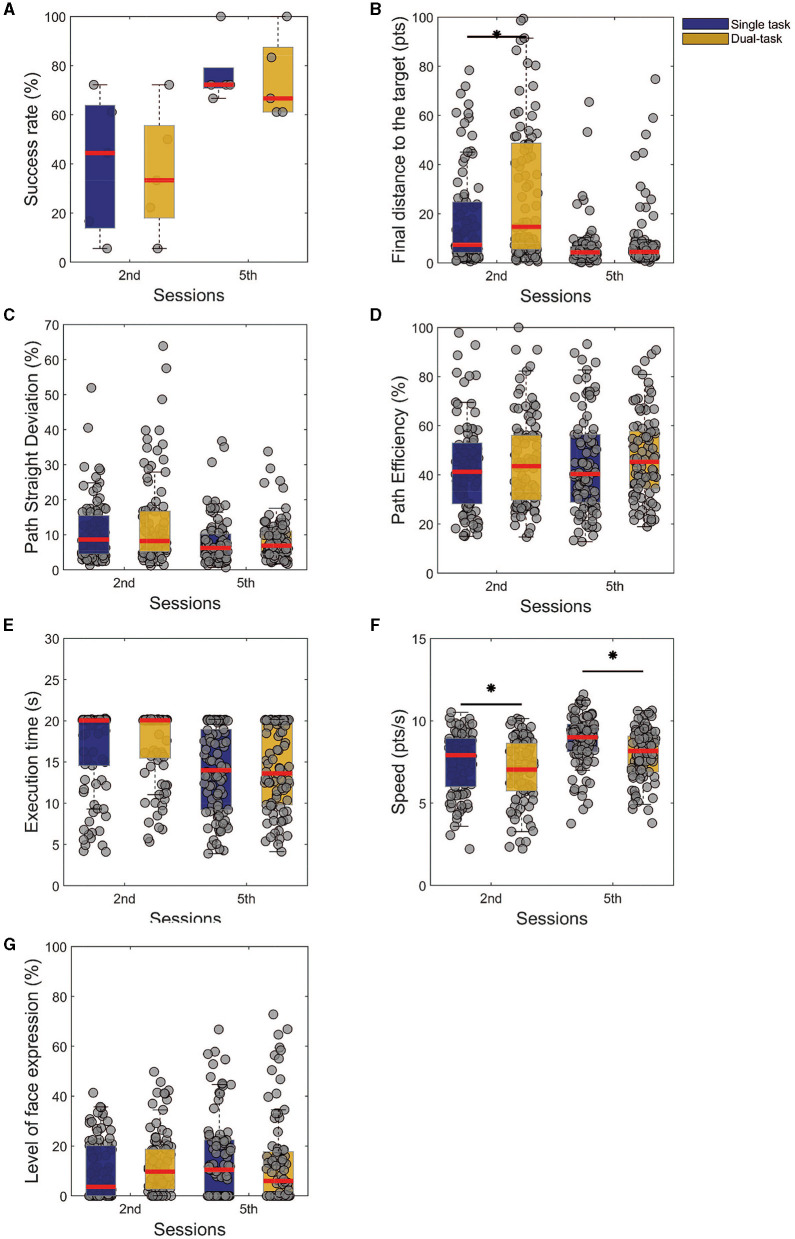
Comparison between the first and last training sessions of single and dual tasks. The presented results are results from Friedman's test analysis with Tukey-Kramer correction, as *post-hoc* (statistical significance *p < 0.05), of a given task among the training sessions. **(A)** Success rate. **(B)** Final distance from the target. **(C)** Path straight deviation. **(D)** Path efficiency. **(E)** Execution time. **(F)** Speed. **(G)** Level of facial expression.

To study the ability of dual tasking, we used the Friedman test to compare the performances of the two tasks for the second and last session, aiming to see if there is any significant difference in the task given a training session, Figures 4A–G. We could observe that the performances were significantly different (Figure 4B, *p* < 0.01, χ2 = 8.76) for the final distance from the target only for the 2nd session, with no effects on the last one. On the other hand, when checking the speed modulation, we note that for both sessions the speed was higher in the single (Figure 4F, *p* < 0.01, χ2 = 14.43). These comparisons show that with the training, no significant differences were found for the assessed measure, except for the speed, which could work as a trade-off to balance the performance. In other words, the subjects were able to control the cursor at a slower speed to not impair their performance on the task.

**Figure 5:**
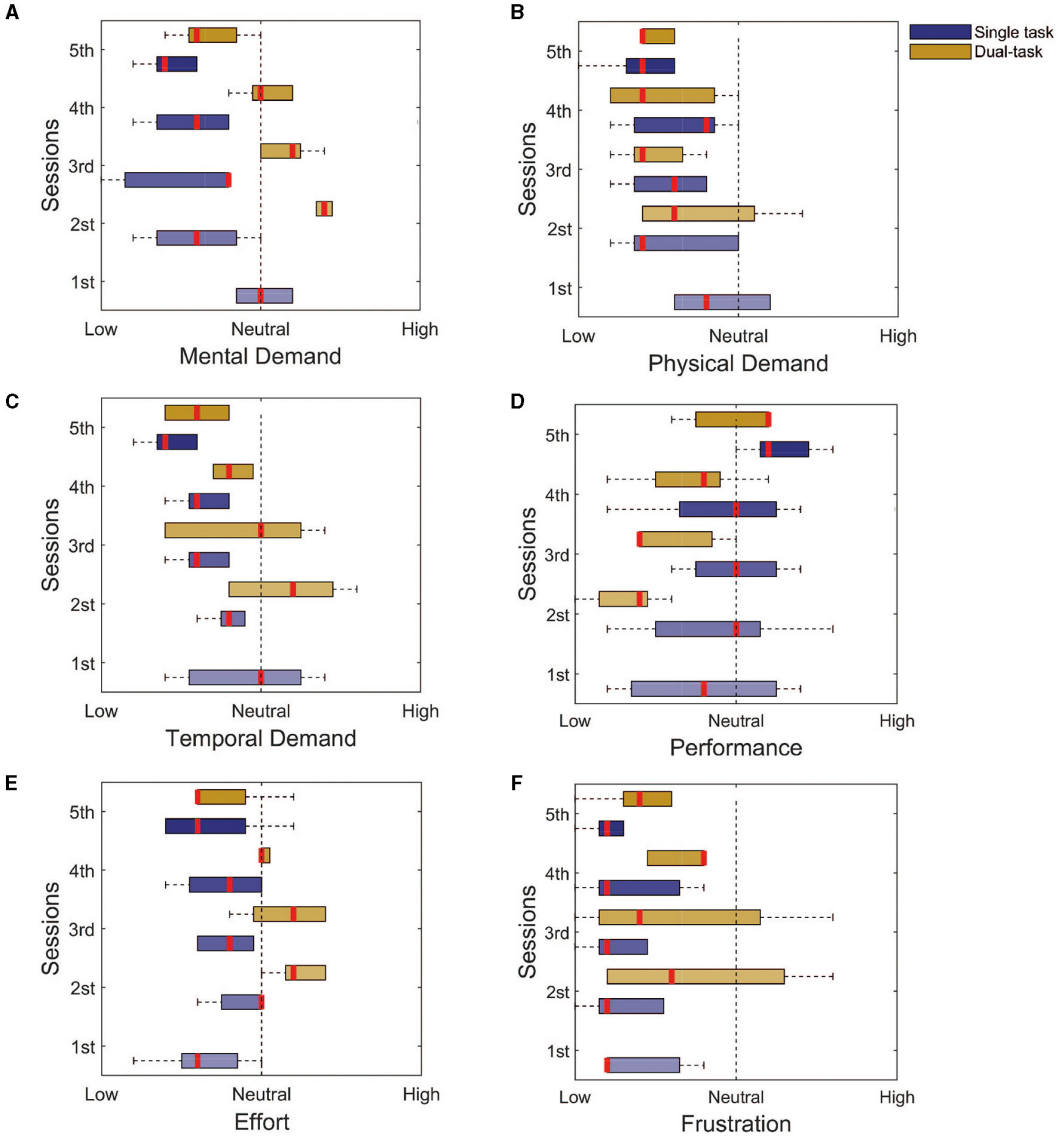
NASA task load index assessment for both the single and dual tasks. **(A)** Mental demand. **(B)** Physical demand. **(C)** Temporal demand. **(D)** Performance. **(E)** Effort. **(F)** Frustration. It is possible to notice that for most of the assessed domains, the response goes toward the lower levels, with the exception of the performance. This finding indicates that the training seems to be enough to reduce the workload associated with the task.

To assess the workload of the tasks, we used the NASA task load questionnaire, in the single and dual-task paradigms. The questionnaire uses 9 levels Likert scale of 1–9 and ranges from 1 = low, 5 = neutral, and 9 = high. For the single task, the participants rated all modalities between neutral and low: the mental demand (Figure 5A, Initial: 5 ± 0; Final: 2 ± 1), physical demand (Figure 5B, Initial: 4 ± 1; Final: 2 ± 1), temporal demand (Figure 5C, Initial: 5 ± 2; Final: 2 ± 1), effort (Figure 5E, Initial: 3 ± 1; Final: 3 ± 1), and frustration (Figure 5F, Initial: 1 ± 0; Final: 1 ± 0). The exception goes to the self-evaluated performance (Figure 5D, Initial: 4 ± 2; Final: 6 ± 1) that started at a low level and went toward higher levels.

For the dual task, the main differences were for the mental demand (Figure 5A, Initial: 7 ± 0; Final: 3 ± 1), temporal demand (Figure 5C, Initial: 6 ± 2; Final: 3 ± 1), and the needed effort (Figure 5A, Initial: 6 ± 1; Final: 3 ± 0), which reduced from a high to a low level with the training. These findings are associated with the improvement in performance (Figure 5D, Initial: 2 ± 1; Final: 6 ± 0). The physical demand (Figure 5B, Initial: 3 ± 1; Final: 2 ± 0), and frustration (Figure 5F, Initial: 3 ± 2; Final: 2 ± 1), from the beginning, was either considered low or neutral.

Another aspect of assessing the dual task is the percentage of error with the visual distractors. The level of error between the real and observed value was always low (Figure 3H), whilst the cursor control performance was improving (Figure 3A). This could indicate a preferred choice of paying attention more to the distractors and over the training, adapting to simultaneously controlling the cursor. Therefore, at the end of the training, subjects were able to do the dual task without significant impairments in terms of performance, except for a different regulation of speed.

## 4. Discussion

We have explored the use of vestigial muscles, which lost their primary function during the evolutionary process (Liugan et al., 2018). Furthermore, we have explicitly controlled the level of facial expression. This aspect represents an advantage over most of the HMI solutions that involve the decoding of head kinematics (LoPresti et al., 2002), tongue movements (Mohammadi et al., 2021), face muscle (Vojtech et al., 2018), or eye tracking (Bissoli et al., 2019), as it does not hinder any of the existing functions but rather provides the users with a new function.

We have shown that with 5 training sessions of <30 min each, all the subjects significantly improved their abilities. We observed similar results between the single-task and dual-task in terms of task workload; in the last training session, all the domains were qualitatively at a low level with the exception of the performance. In terms of the evaluated performance, we found similar results on the last day for the single and dual tasks for the success rate, path straight deviation, path efficiency, execution time, and final distance to the target.

Compared to the available options in the literature using the AM, our method provides the user the possibility to stop and control in an easy manner, modulate the speed of the end-effector, and independently control each one of the axes. In relation to Schmalfuß et al. (2016), our solution differs from using a non-invasive recording approach for acquiring the EMG signals. This feature can enhance the usability and reduce the risks associated with invasive procedures. Additionally, each axis of our system can be individually controlled, providing an extra degree of freedom in movements that can improve the accuracy and precision of the control. When comparing to solutions that use only one AM to control both DoF, we have observed that in such cases, the user is required to either modulate the spectral features of the recorded sEMG (Perez-Maldonado et al., 2010), which can be challenging and demanding more training, or the 2 DoF problem is simplified by 1 DoF, which may be limiting in non-virtual applications (O'Meara et al., 2019). In contrast, our proposed solution shows an advantage in terms of control performance and usability. The user can control each axis separately and with high accuracy, without being burdened by complex modulation tasks or restricted by a reduced DoF. We believe that these aspects, together with the short training needed to achieve a good level of control, are the highlights of this approach and what make viable solutions for HMI applications.

One of the limitations of our study is that we did not evaluate different approaches that could be used to control the cursor with the activation of the auricular muscle to assess the most intuitive one. The comparison with the existing literature is also complicated since different measurements and paradigms are used to assess the HMI. However, our main goal was to provide independent control over the axes and enable movement in all four directions without adding another state to the state machine used for the blocking mechanism. We believe that this mechanism, together with the penalty for facial expressions, was a key factor in our development. Through a few training sessions, we were able to demonstrate that all subjects could control the HMI and reduce their reliance on facial expressions. The locking mechanism also introduced a novelty that allowed for more independence in control, enabling users to move the cursor freely in both directions, control it in only one direction, or stop it at the desired position without worrying about the contraction of the AM.

In an HMI for bidimensional steering control based on muscle contraction, a blocking mechanism plays a crucial role in ensuring the safety and accuracy of the system. Without a blocking mechanism, the system would react to any muscle contraction, whether intentional or not, potentially causing unintended movements of the end-effector. The blocking mechanism acts as a filter, allowing only intended muscle contractions to be translated into cursor movements.

Is worth emphasizing that some current implementations with AM use a threshold of minimum contraction as a relax state that does not interfere with the movement (Perez-Maldonado et al., 2010; Schmalfuß et al., 2016; O'Meara et al., 2019), and although these muscle relaxations may not produce any movement or command in the interface, they can still create noise in the system and interfere with its accuracy, by oscillating the current position.

To ensure an objective and consistent evaluation of the performance of our HMI of cursor control, we used only one difficulty level according to Fitt's law. We acknowledge the practice in the literature of using several IDs for a broader exploration of the HMIs transfer rates considering the different conditions, but here we wanted to focus more on only one to evaluate the interface's effectiveness without any other confounding factors that might influence the user's performance. Even though, regarding other applications for cursor control using different modalities recording modalities, we can cite Vojtech et al. (2020); for example, that presented a hybrid HMI that combines head kinematics and EMG recordings for 2 DoF cursor control. The accuracy of the system reached 70.5% in one of the tested conditions. Rahmaniar et al. (2022); on the other hand, proposed a head gesture recognition interface for cursor control that also achieved high target selection scores. Chin (2008) showed in a study of with HMIs that compared the use of EMG, eye tracking, and a combination of both as solutions for cursor control for users with motor disabilities that, although the hybrid solution has a slower control than eye-tracking only, it was more reliable for click operations. Therefore, we believe that our results show a competitive performance of the HMI of cursor control, with a success rate of 72.22 ± 6.67%. Moreover, our solution may provide an alternative to these more established solutions as an application that uses a biological source that is not directly involved in other essential functions, due to the AM being a vestigial muscle.

This study was the first step in developing the HMIs clinical population with motor disabilities. Our solution could be applied, for example, with SCI people to control a wheelchair, a cursor on computers or smartphones, or with people with amputation to control a prosthesis. A particularly interesting evolution of this approach can also be in the field of human augmentation with a population with no motor impairment. The AM control could allow for controlling extra limbs or end-effectors (Dominijanni et al., 2021).

Further studies will be necessary to investigate how much of the contractions elicited by the AM are independent of the adjacent muscles, and if it is the case, proper training to reduce that can be proposed using biofeedback techniques to enhance motor learning of muscles (Perez-Maldonado et al., 2010; Rodrigues et al., 2022; Moreira et al., 2023). Also, we made the choice to keep the current protocol short, but we can notice that by the end of the 5^th^ session, participants had not reached a plateau, and further improvement could be possible with longer use of the AM controller. Tests in a real-world environment/application with subjects of a wider age range and with and without motor impairments can also help to understand the extension for applications of this solution since this variability can have a direct impact on motor learning (Jongbloed-Pereboom et al., 2019), but also elicit new strategies on how to facilitate the training phase.

## Data availability statement

The raw data supporting the conclusions of this article will be made available by the authors, without undue reservation.

## Ethics statement

The study was approved by the Commission Cantonale d'éthique de la recherche Genève (BASEC-ID: 2019-02176). The patients/participants provided their written informed consent to participate in this study. Written informed consent was obtained from the individual(s) for the publication of any potentially identifiable images or data included in this article.

## Author contributions

DP contributed to the conception and design of the work, acquisition, analysis, interpretation of data, writing, and review of the article. JF contributed to the data interpretation and review of the article. SM contributed to the conception of the work and writing and review of the article. SS contributed to the conception and design of the work, data analysis and interpretation, writing, and review of the article. All authors contributed to the article and approved the submitted version.
