# Supplementary material for: Human-machine interface for two-dimensional steering control with the auricular muscles
Source: Front Neurorobot. 2023 Jun 5;17:1154427. doi: 10.3389/fnbot.2023.1154427 (PMC10277645; doi:10.3389/fnbot.2023.1154427)
Supplement: Supplementary file 5 [file Data_Sheet_1.PDF]

Table S1: Individual success rate throughout the training for the single-task – Cohen's d = 1.81 (1st and 5th session; large effect size)

	Sessions					
Subjects		1st	2nd	3rd	4th	5th
	S01	19.44%	5.56%	22.22%	33.33%	72.22%
	S02	55.56%	44.44%	66.67%	77.78%	100.00%
	S03	52.78%	61.11%	61.11%	55.56%	72.22%
	S04	2.78%	16.67%	33.33%	27.78%	66.67%
	S05	58.33%	72.22%	66.67%	61.11%	72.22%

Table S2: Individual final distance from the target throughout the training for the single-task (mean ± standard error in points) – Cohen's d = 1.75 (1st and 5th session; large effect size)

	Sessions					
Subjects		1st	2nd	3rd	4th	5th
	S01	34.48 ± 4.57	50.02 ± 11.44	33.15 ± 5.88	16.93 ± 3.82	9.01 ± 2.93
	S02	19.61 ± 3.52	17.56 ± 4.99	15.41 ± 5.09	6.44 ± 1.54	3.80 ± 0.68
	S03	15.80 ± 3.11	14.68 ± 4.43	14.89 ± 4.63	21.45 ± 5.35	6.96 ± 1.59
	S04	32.56 ± 4.80	21.72 ± 4.25	17.32 ± 3.60	22.41 ± 5.19	8.99 ± 3.55
	S05	8.31 ± 1.47	8.32 ± 2.35	10.83 ± 3.49	3.65 ± 0.40	3.47 ± 0.41

Table S3: Individual success rate throughout the training for the dual-task – Cohen's d = 3.53 (2st and 5th session)

	Sessions				
Subjects		2nd	3rd	4th	5th
	S01	5.56%	22.22%	55.55%	61.11%
	S02	50.00%	55.56%	83.33%	83.33%
	S03	33.33%	50.00%	44.44%	66.67%
	S04	22.22%	38.89%	50.00%	61.11%
	S05	72.22%	55.56%	72.22%	100.00%

Table S4: Individual final distance from the target throughout the training for the dual-task (mean ± standard error in points) – Cohen’s d = 1.13 (1st and 5th session; large effect size)

		Sessions			
**Subjects**		2nd	3rd	4th	5th
	S01	63.37 ± 9.33	33.52 ± 5.95	6.25 ± 1.20	7.84 ± 1.74
	S02	25.85 ± 7.54	14.18 ± 4.39	7.90 ± 3.51	8.37 ± 3.47
	S03	22.94 ± 5.28	16.38 ± 5.84	21.88 ± 6.34	9.96 ± 4.00
	S04	29.29 ± 6.06	23.80 ± 5.54	12.26 ± 3.27	13.85 ± 3.78
	S05	11.51 ± 3.20	11.04 ± 3.19	4.26 ± 1.06	4.23 ± 0.45
