# Supplementary material for: Human-machine interface for two-dimensional steering control with the auricular muscles
Source: Front Neurorobot. 2023 Jun 5;17:1154427. doi: 10.3389/fnbot.2023.1154427 (PMC10277645; doi:10.3389/fnbot.2023.1154427)
Supplement: Supplementary file 6 [file Image_1.pdf]

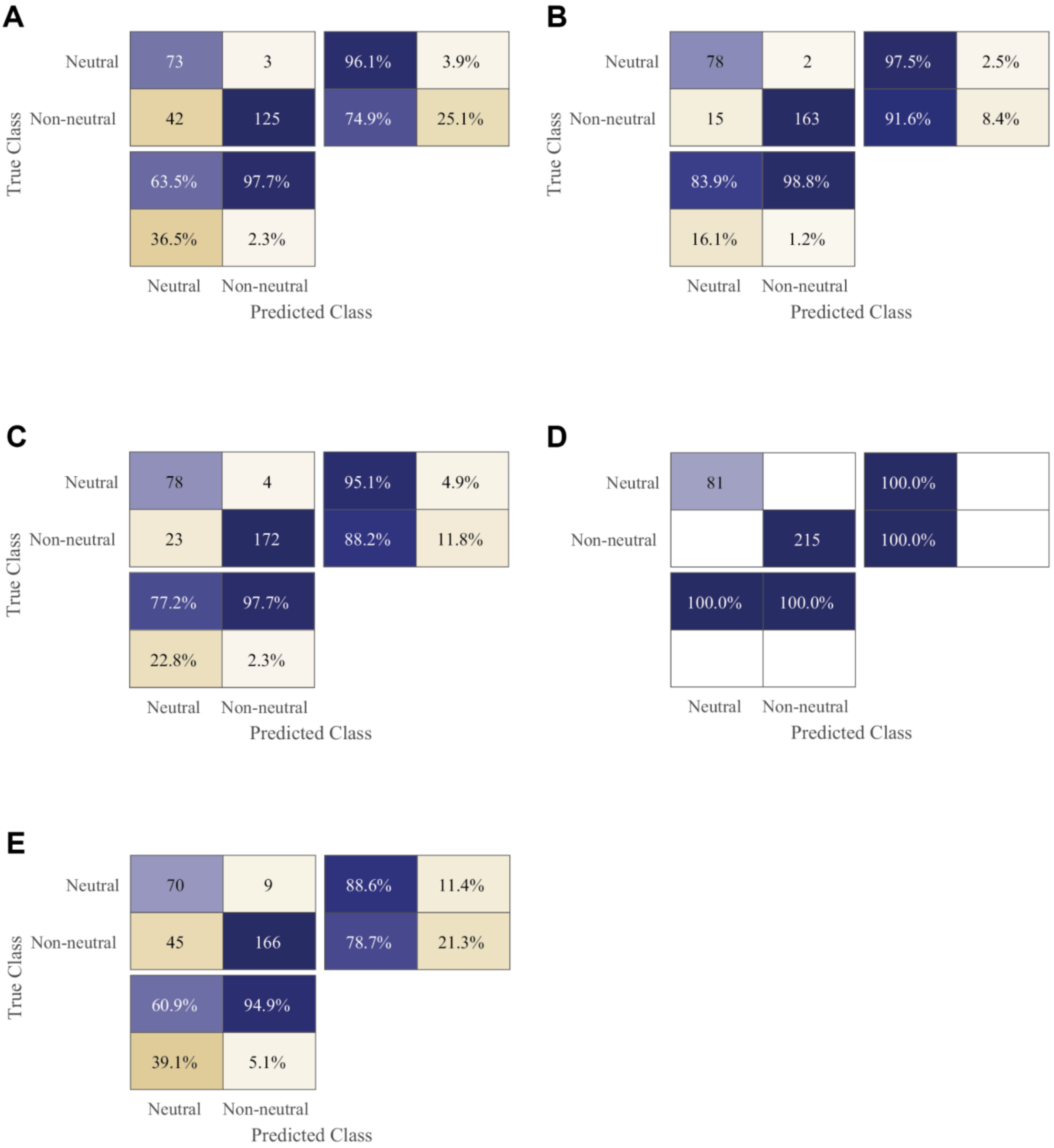

Figure S1: Confusion matrix of the linear discriminant models used to discriminate the neutral and non-neutral facial expressions for each subject. The models were trained for each session and subject separately, using the features and protocol explained in the methods sessions. To assess the overall performance of the models we used 80% of the data for its training and 20% to test it. The matrix accounts for the model performances of all sessions given a subject, for better summarization. (A-E) Confusion matrix with model's performance for each participant of the experiment. We can note very high accuracy rates in detecting the non-neutral state (between 94.5% and 100% accuracy) and a lower for the neutral class (60.9% to 100 %);
